# A 13-year-old caucasian boy with cleidocranial dysplasia: a case report

**DOI:** 10.1186/1756-0500-6-6

**Published:** 2013-01-05

**Authors:** Olga-Elpis Kolokitha, Ioulia Ioannidou

**Affiliations:** 1 Department of Orthodontics, School of Dentistry, Aristotle University of Thessaloniki, Thessaloniki, GR - 54124, Greece

**Keywords:** Cleidocranial dysplasia, Delayed eruption, Supernumerary teeth

## Abstract

**Background:**

Cleidocranial dysplasia (CCD) is a rare congenital autosomal dominant skeletal disorder. The disorder is caused by heterozygosity of mutations in human *RUNX2*, which is present on the short arm of chromosome 6p21*.* The incidence of CCD is one per million births. CCD appears spontaneously with no apparent genetic cause in approximately 40% of affected patients, and one in three patients has unaffected parents. The most prevalent features associated with CCD are aplastic or hypoplastic clavicles, supernumerary teeth, failed eruption of permanent teeth, and a hypoplastic maxilla.

**Case presentation:**

A 13-year-old Caucasian boy presented with a chief complaint of delayed eruption of the permanent anterior teeth. The patient was subsequently diagnosed with CCD based on the clinical examination, panoramic X-ray, anterior-posterior and lateral cephalogram, and chest radiograph findings. The details of this case are herein reported because of the extremely low incidence of this disorder.

**Conclusions:**

CCD is of clinical importance in dentistry and medicine because it affects the bones and teeth and is characterized by many changes in skeletal patterning and growth. Particularly in dentistry, CCD is of great clinical significance because is associated with delayed ossification of the skull sutures, delayed exfoliation of the primary teeth, lack of permanent teeth eruption, multiple supernumerary teeth, and morphological abnormalities of the maxilla and mandible. Patients with CCD seek treatment mainly for dental problems. Knowledge of the pathogenesis, clinical characteristics, and diagnostic tools of CCD will enable clinicians to render the appropriate treatment to improve function and aesthetics. Early diagnosis of CCD is crucial for timely initiation of an appropriate treatment approach.

## Background

Cleidocranial dysplasia (CCD) is a rare congenital skeletal disorder that affects the bones and teeth and shows an autosomal inheritance pattern. The disorder is characterized by hypoplasia or aplasia of the clavicles, delayed closure of the fontanelles, supernumerary teeth, short stature, and other changes in skeletal patterning and growth [1-3].

Hereditary transmission of this syndrome was reported many years ago; however, the etiology and pathogenesis of the syndrome has remained unknown for many decades. Genetic studies of individuals from families bearing the syndrome, as well as experimental studies on transgenic mice, have presented new data regarding the syndrome’s clinical manifestation and type of hereditary transmission. The responsible gene for the pathogenesis of CCD has been mapped on the short arm of chromosome 6p21, core binding factor a-1 (*Cbfa1*) [4]. Sixteen different types of mutations on various areas of the *Cbfa1* gene have been detected and are correlated with the different clinical manifestations of the syndrome. With these data, specific areas and sequences implicated in the appearance of mild clinical characteristics to severe skeletal manifestations, as well as individualized dental anomalies, have been mapped in great detail. The *Cbfa1* (*RUNX2*) gene controls normal development and growth of the human skeleton through the progression of intramembranous and endochondral ossification, and may be related to delayed ossification of the skull, teeth, pelvis, and clavicles [5,6]. *Cbfa1* also regulates the gene expression of dental epithelium mesenchymal cells, and in this manner, the deficiency of transcription factor Cbfa1 leads to the manifestation of the dental anomalies observed in patients with CCD [7]. A study of the cellular mechanisms of dental eruption in heterozygous *Cbfa1*+/- test animal subjects revealed a decreased number of osteoclasts that contribute to normal resorption of the alveolar bone during tooth eruption. The decreased number of osteoclasts in patients with CCD leads to delayed eruption and increased numbers of impacted teeth [8]. In conclusion, the deficiency of transcription factor Cbfa1 in CCD leads to deregulation of the morphogenetic mechanisms of skeletal and dental development and growth.

Genotype-phenotype correlations have revealed quantitative dependency of the severity of skeletal and dental development on the type of mutations in the *RUNX2* gene. The type and location of specific mutations within the *RUNX2* gene impact the expressivity of the CCD phenotypic findings, thus correlating the genetic variations in the corresponding gene with the clinical manifestations of the syndrome [9].

According to the literature, the most prevalent features associated with CCD are supernumerary teeth, failed eruption of permanent teeth, a hypoplastic maxilla, and aplastic or hypoplastic clavicles [1,2].

The incidence of CCD is one per million births. CCD appears spontaneously with no apparent genetic cause in approximately 40% of affected patients, and one in three patients has unaffected parents [3,10]. We herein report a case of CCD that was not previously diagnosed because of the extremely low incidence of this disorder.

## Case presentation

A 13-year-old Caucasian boy was referred to the orthodontic clinic of the School of Dentistry, Aristotle University, Thessaloniki, Greece. He presented with his father with the chief complaint of delayed eruption of the permanent anterior teeth. Because of this delay, he was often teased at school. He was told that his smile was very unpleasant, which resulted in difficulty communicating with his classmates. His dentist could not explain the delayed eruption of his anterior teeth. Failed eruption of his permanent teeth was the reason that the patient sought treatment. His parents reported an unremarkable medical history. He was in good health with no medications and no known allergies. His dental history revealed no trauma to the mouth, teeth, or jaws.

**Figure 1:**
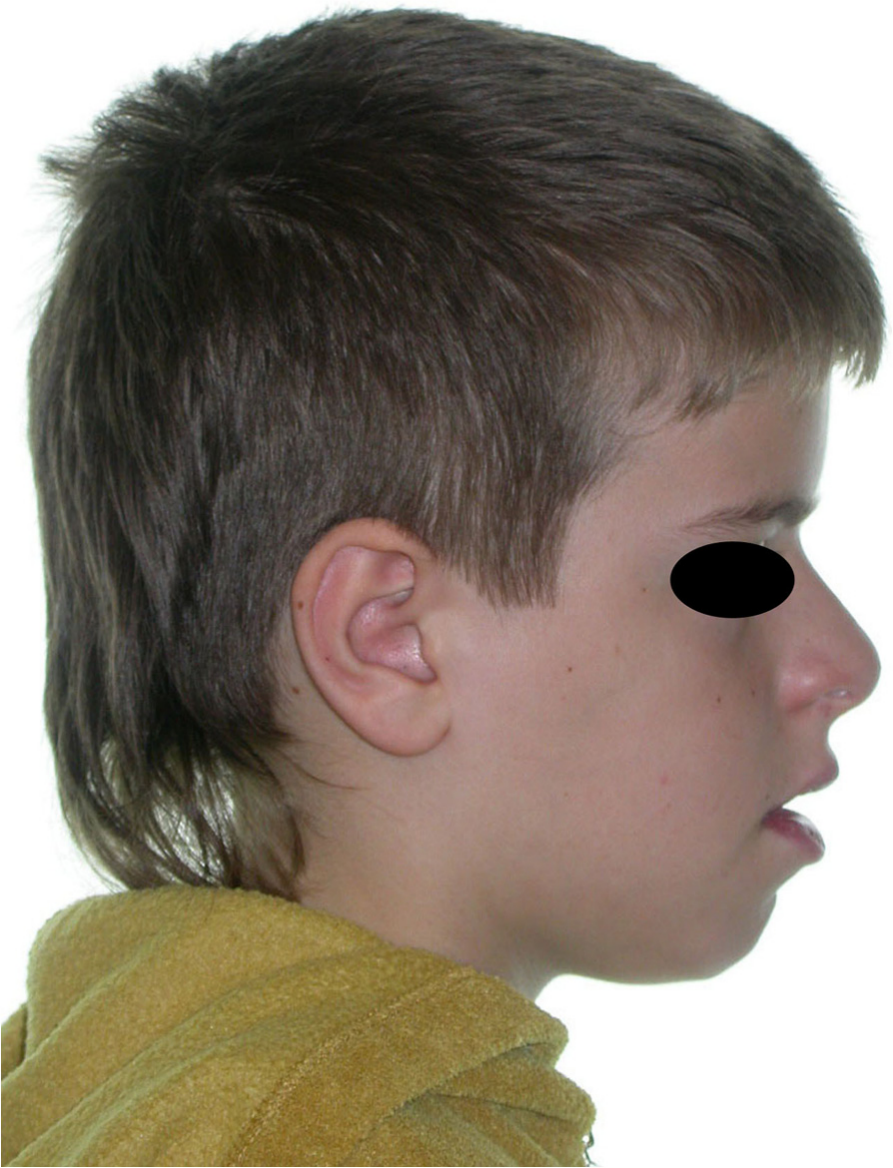
Facial view showing bulging calvaria, depressed nasal bridge, and hypoplastic maxillary and zygomatic bones.

**Figure 2:**
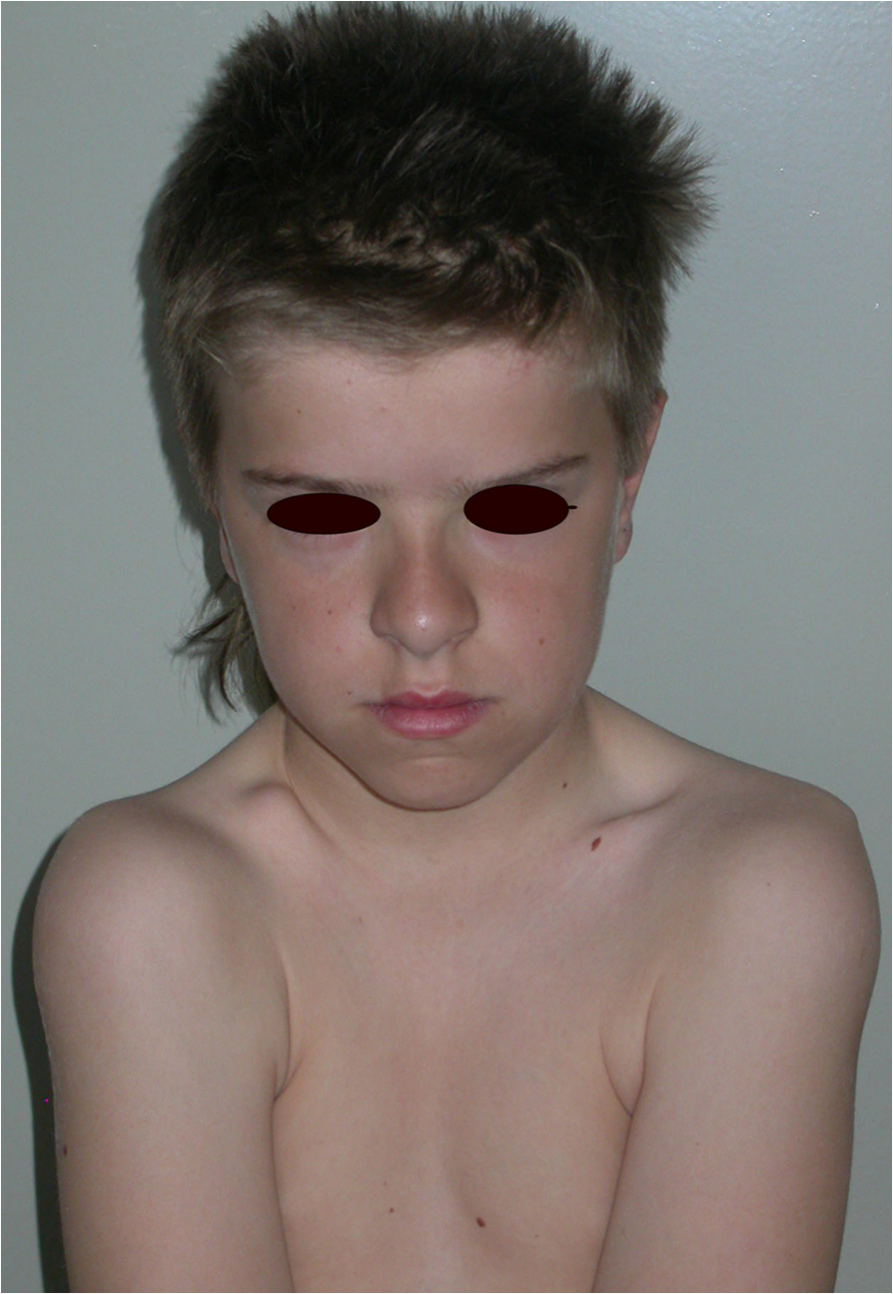
**Facial view showing a brachycephalic head and hypermobility of the shoulders.** (From Kolokitha OE, Papadopoulou AK [1] with permission).

**Figure 3:**
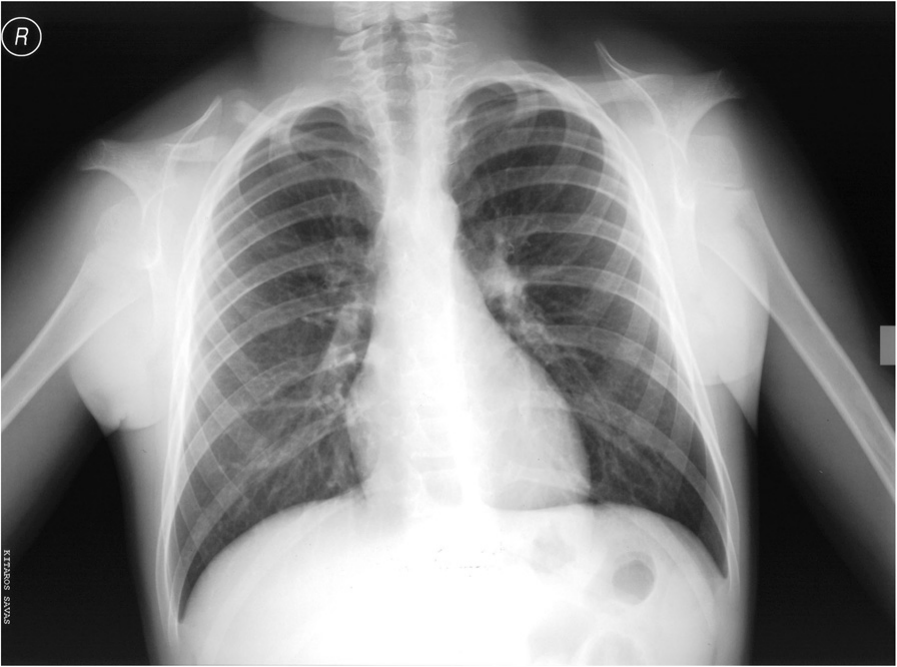
**Chest radiograph showing hypoplastic clavicles.** (From Kolokitha OE, Papadopoulou AK [1] with permission).

**Figure 4:**
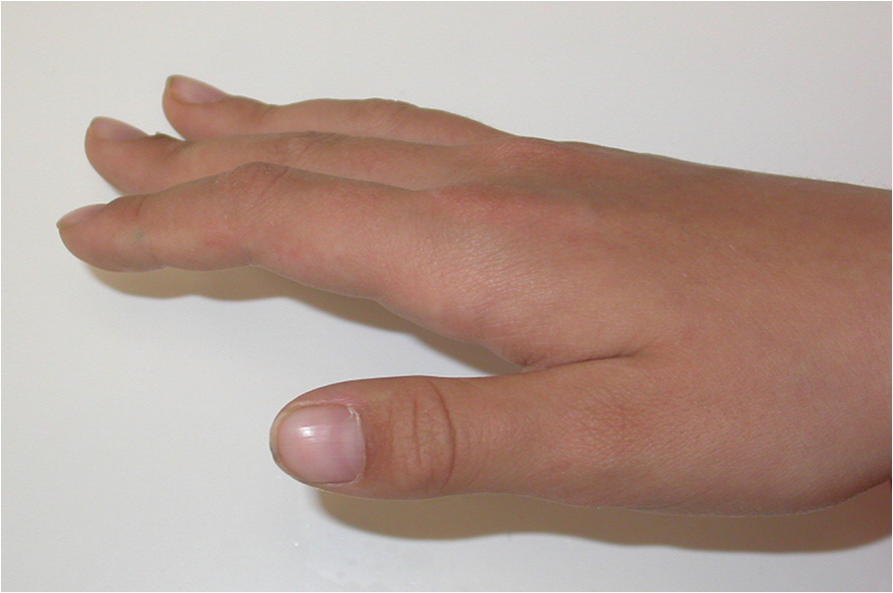
Hand photo showing finger abnormalities.

**Figure 5:**
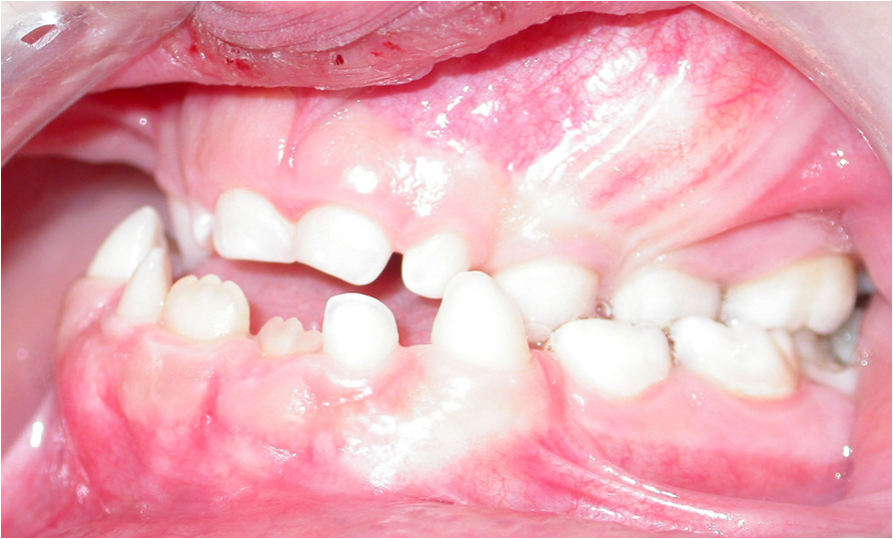
Intraoral view showing persistence of primary dentition, class III malocclusion, and posterior crossbite.

A general physical examination revealed a short-statured, well-oriented young boy with narrow, drooped shoulders. Facial examination showed a brachycephalic head with frontal and parental bossing, hypoplastic maxillary and zygomatic bones, bulging calvarium, and depressed nasal bridge with a broad alar base (Figure 1). Further examination showed an abnormal facility in opposing his shoulders (Figure 2) due to malformed or absent clavicles, which was later confirmed with chest radiographs (Figure 3). A short middle phalanx of the fifth finger and curved nails were also noted (Figure 4). Intraoral examination revealed persistence of the primary dentition, delayed eruption of the permanent teeth, an Angle class III malocclusion, negative overjet, bilateral posterior crossbite (Figure 5), and high arched palate.

**Figure 6:**
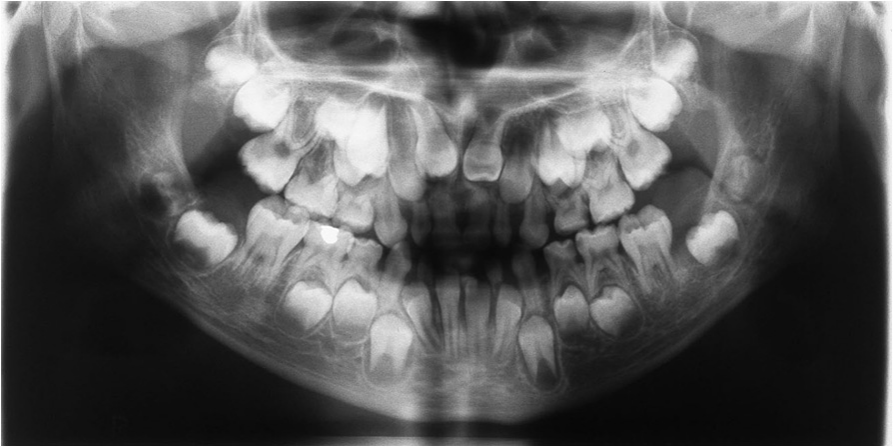
**Panoramic X-ray showing supernumerary teeth and impaction of the upper anterior teeth.** (From Kolokitha OE, Papadopoulou AK [1] with permission).

**Figure 7:**
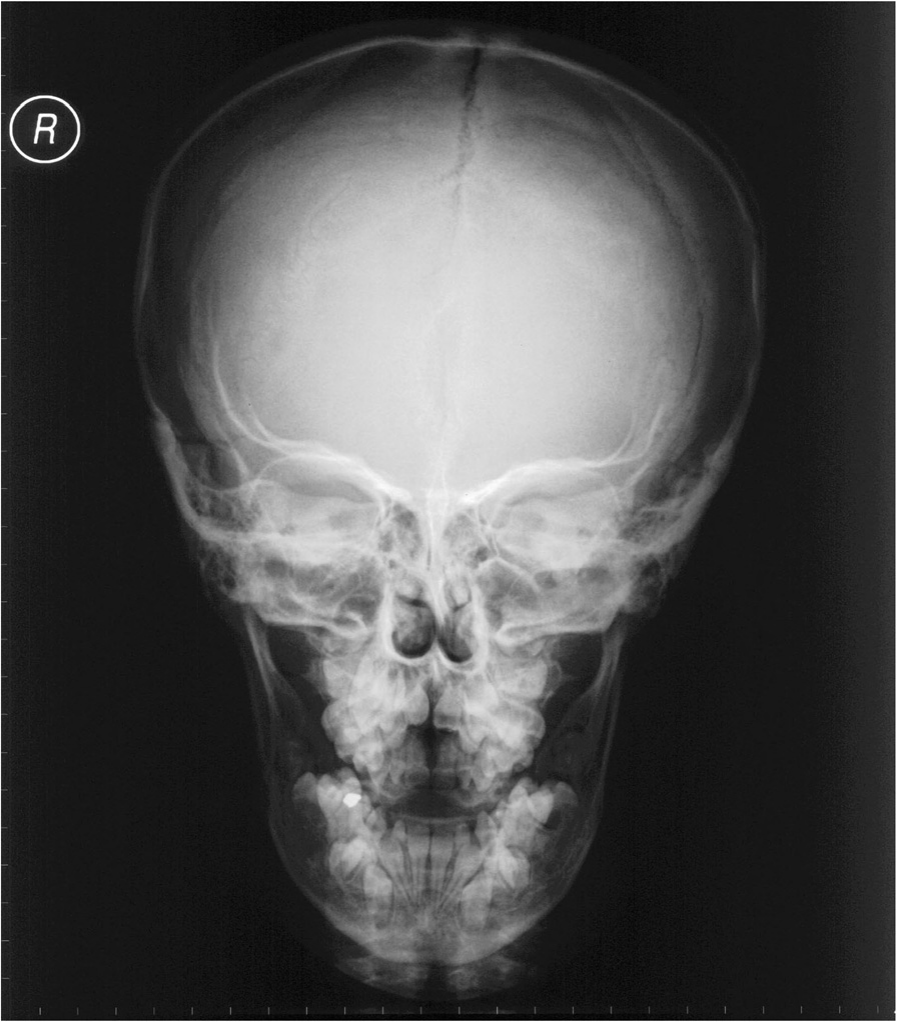
**Anteroposterior cephalogram showing open sutures of the skull, small maxillary sinuses, and missing nasal bone.** (From Kolokitha OE, Papadopoulou AK [1] with permission).

**Figure 8:**
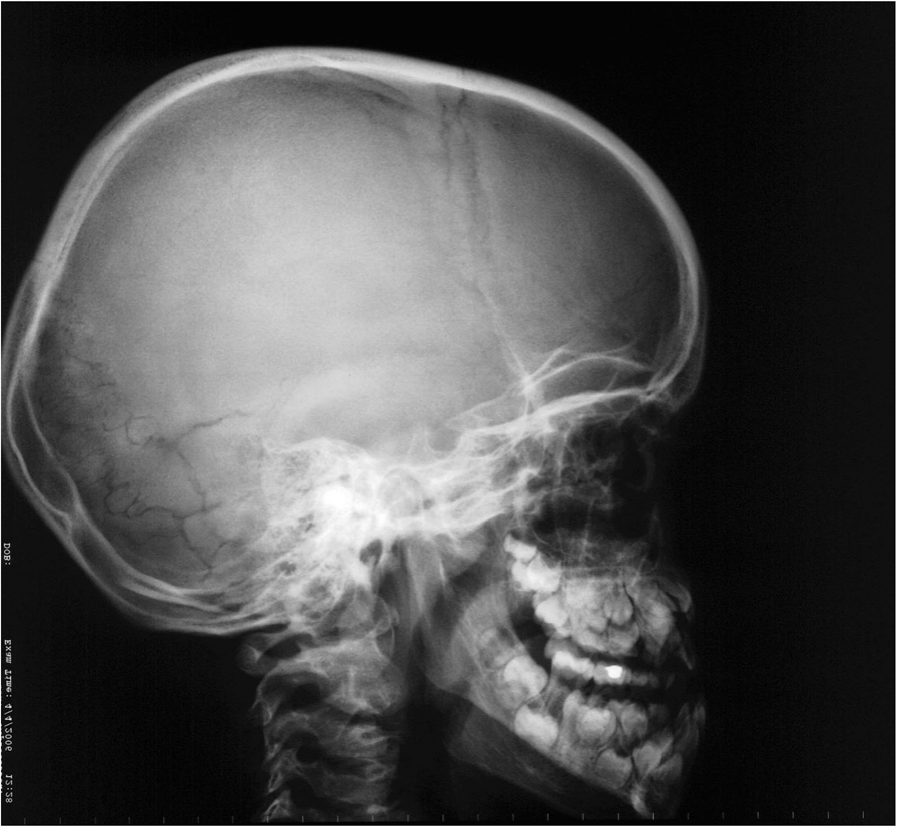
**Lateral cephalometric X-ray showing numerous Wormian bones, large fontanelles, hypoplastic maxilla, and prognathic mandible.** (From Kolokitha OE, Papadopoulou AK [1] with permission).

A panoramic X-ray showed the presence of two supernumerary teeth in the area of the maxillary incisors, impaction of the anterior maxillary teeth, and caries in several teeth (Figure 6). The anterior-posterior (Figure 7) and lateral cephalogram (Figure 8) showed open sutures of the skull, large fontanelles, small maxillary sinuses, numerous Wormian bones in the lambdoidal sutures, a missing nasal bone, a hypoplastic maxilla, and a prognathic mandible. The cephalometric analysis confirmed the class III skeletal malocclusion as evidenced by the combination of an underdeveloped maxilla and large mandibular body. The anterior and posterior cranial base was significantly reduced and had a reduced angle of flexure. Increased horizontal mandibular growth was found along with anterior rotation of the mandible in relation to the cranial base. The patient had deficient growth of the midface and decreased lower facial height. The lower incisors were retroclined, indicative of class III malocclusion. The chest radiograph showed a very narrow thorax with oblique ribs and hypoplastic clavicles (Figure 3).

**Figure 9:**
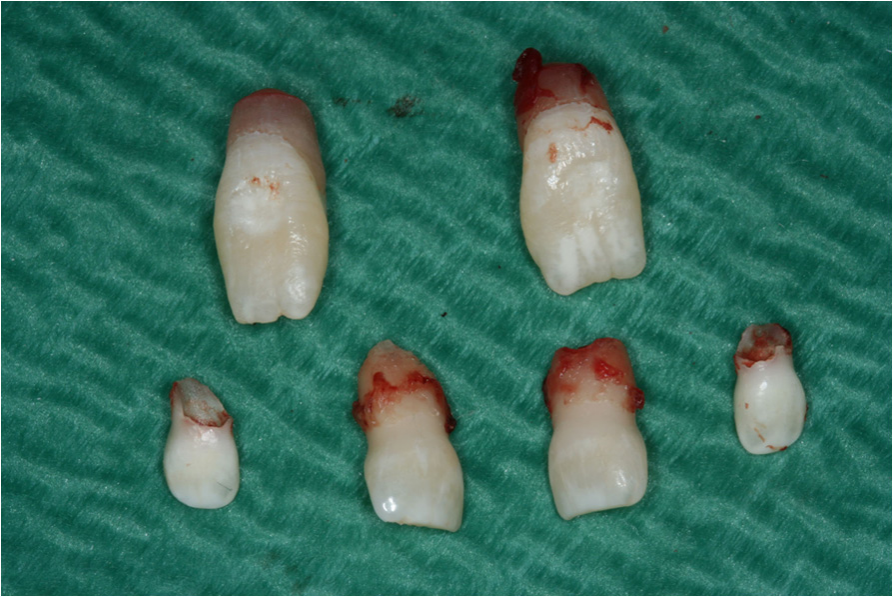
Extraction of deciduous and supernumerary teeth.

**Figure 10:**
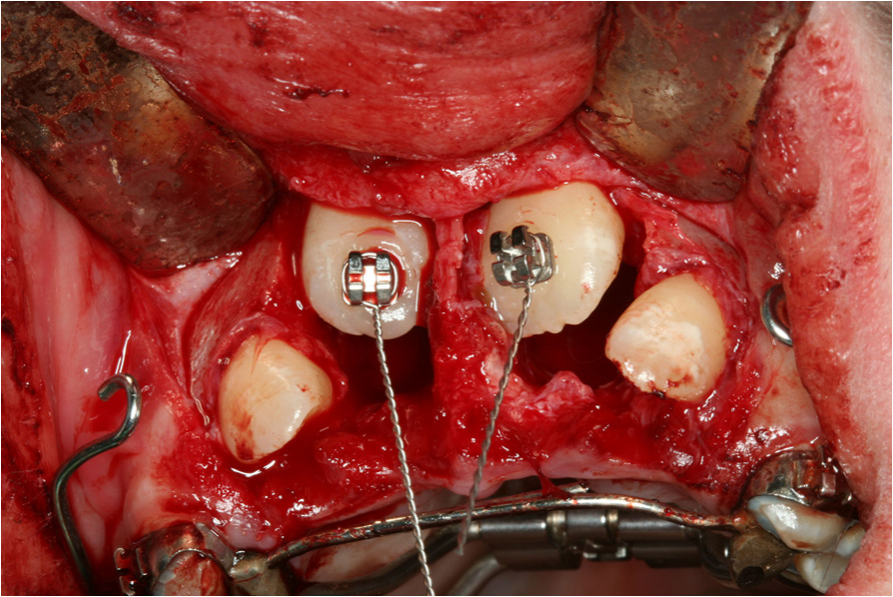
Surgical exposure of impacted teeth and bracket bonding for orthodontic traction of permanent teeth.

A diagnosis of CCD was made based on the clinical and radiological examination findings. The oral manifestations in this patient were class III skeletal malocclusion due to a combination of an underdeveloped maxilla and mandibular pragmatism, bilateral posterior crossbite, high arched palate, retained deciduous teeth, two supernumerary teeth, and impaction of the anterior permanent teeth. The treatment plan comprised orthodontic treatment for the skeletal malocclusion with Hyrax appliance and Delaire mask extraction of the deciduous teeth and supernumerary teeth (Figure 9) and surgical exposure and orthodontic traction of the permanent teeth (Figure 10). The goal of treatment was to establish a functional occlusion and obtain an aesthetic result.

Because this patient had not been previously diagnosed, a molecular genetic analysis was proposed. The patient’s parents declined molecular genetic analysis for personal reasons; therefore, identification of the responsible gene was not possible.

Aplastic or hypoplastic clavicles is a characteristic and pathognomonic feature of CCD that is responsible for the appearance of narrow, drooped shoulders and for the wide range of shoulder movement, resulting in the ability of the patient to approximate his or her shoulders in front of the chest [2,11,12]. This ability is not always recognized by the patient, nor are the parents always aware of it, as was true in the present case.

The skull base is dysplastic with reduced growth, resulting in increased skull width and leading to brachycephaly and hypertelorism that are usually associated with frontal and biparietal bone bossing [2]. Midface deficiency; underdeveloped paranasal sinuses; hypoplastic maxillary, nasal, and zygomatic bones; recession of the nasal bridge; a wide alar base; and prominent frontal, parietal, and occipital bones are common findings in patients with CCD. The underdeveloped maxilla with overall deficient growth of the midface combined with the direction of mandibular condylar growth and its anterior rotation give the impression of relative or actual mandibular prognathism [2,13,14]. In the present case, the cephalometric analysis showed deficient growth of the midface along with a large mandibular body, which confirmed actual mandibular prognathism; increased horizontal mandibular growth; and an anterior rotation of the mandible, resulting in a skeletal class III malocclusion.

Dental abnormalities are typical main features of CCD, and they occur in 93.5% of affected patients [15]. The primary dentition usually develops relatively normally [16], while the permanent dentition is severely disturbed. Supernumerary teeth, prolonged retention of the primary dentition, failed eruption of the permanent teeth, multiple crown and root abnormalities, crypt formation around impacted teeth, and ectopic locations of teeth are the more common dentition disturbances [11]. A highly arched palate is a less common oral manifestation of CCD. In the present case, failed eruption of the permanent teeth was the reason that the patient sought treatment. He presented with multiple supernumerary teeth that impeded normal eruption of the permanent teeth.

Other features seen in CCD are deformities of the thoracic region, pelvic and pubic bones, and fingers. More specifically, finger abnormalities include short, tapered fingers and anomalies of the phalangeal, tarsal, metatarsal, carpal, and metacarpal bones [7,10,17,18].

CCD is highly polymorphic; therefore, a radiological study is necessary to establish a reliable diagnosis [10,12,13,15,16]. A complete radiological examination includes panoramic, skull, and chest radiographs. Absolute verification of the diagnosis of CCD can only be obtained by molecular genetic analysis [1-3].

A diagnosis of CCD was made for the first time in this patient and was based on the clinical facial and oral examination, panoramic X-ray, anterior-posterior and lateral cephalogram, and chest radiograph findings.

The clinical findings of CCD, although present at birth, could be easily missed because of their extremely low frequency, rare manifestation of the typical extraoral symptoms in early childhood, and the clinical variety of the disorder [3], as was true in the present case. Because the most striking dental sign is the failure of eruption of the upper frontal permanent teeth, patients with CCD are often not recognized before the second dentition. Early diagnosis is crucial to timely initiation of an appropriate treatment approach [3,19]. In addition, molecular genetic analysis is a very useful diagnostic tool for early detection when CCD is suspected. The successful treatment of patients with CCD requires a team approach and a compliant patient. An interdisciplinary treatment approach involving orthodontics, maxillofacial surgery, and prosthodontics is obligatory. Genetic counseling for family planning should certainly be advised. Good collaboration among the specialists, the patient, and the patient’s family is essential for an organized treatment approach in which each member can contribute his or her expertise for the best treatment outcome [1,12]. The overall treatment goal is to establish functional occlusion and an aesthetic facial and dental appearance. Sometimes this disorder causes psychological problems for the patients; therefore, proper motivation and support are important.

## Conclusions

Knowledge of the clinical characteristics, family history, and diagnostic tools for CCD will enable the clinicians to achieve an early diagnosis and implement appropriate treatment to improve function and aesthetics. Molecular genetics analysis can provide an absolute verification of the diagnosis of CCD and allow for identification of the responsible gene.

## Consent

Written informed consent was obtained from the patient’s father for publication of this case report. A copy of the written consent is available for review by the Editor-in-Chief of this journal.

## Competing interests

The authors declare that they have no competing interests.

## Authors’ contributions

OEK and II analyzed and interpreted the patient’s data regarding the associated dental problems. OEK was a major contributor in writing the manuscript. OEK and II jointly performed the treatment procedures. All authors read and approved the final manuscript.
